# Next-generation sequencing reveals additional HLA class I and class II alleles associated with type 1 diabetes and age at onset

**DOI:** 10.3389/fimmu.2024.1427349

**Published:** 2024-08-09

**Authors:** Antonietta Robino, Elena Bevilacqua, Luana Aldegheri, Andrea Conti, Valentina Bazzo, Gianluca Tornese, Eulalia Catamo

**Affiliations:** ^1^ Institute for Maternal and Child Health – IRCCS Burlo Garofolo, Trieste, Italy; ^2^ Transfusion Medicine Department, Azienda Sanitaria Universitaria Giuliano Isontina, Trieste, Italy; ^3^ Department of Medical Sciences, University of Trieste, Trieste, Italy

**Keywords:** age at onset, human leukocyte antigen, next generation sequencing, pediatric, type 1 diabetes

## Abstract

**Introduction:**

Type 1 diabetes is an autoimmune disease with an significant genetic component, played mainly by the *HLA* class II genes. Although evidence on the role of *HLA* class I genes in developing type 1 diabetes and its onset have emerged, current *HLA* screening is limited to determining DR3 and DR4 haplotypes. This study aimed to investigate the role of *HLA* genes on type 1 diabetes risk and age of onset by extensive typing.

**Methods:**

This study included 115 children and young adults with type 1 diabetes for whom typing of *HLA-A*, *-B*, *-C*, *-DRB1*, *-DRB3/4/5*, *-DQA1*, *-DQB1*, *-DPA1* and *-DPB1* genes was conducted using Next Generation Sequencing.

**Results:**

We observed that 13% of type 1 diabetes subjects had non-classical *HLA* haplotypes that predispose to diabetes. We also found that compared to type 1 diabetes subjects with classical *HLA* haplotypes, non-classical *HLA* subjects had a significantly higher frequency of HLA-B*39:06:02 (p-value=0.01) and HLA-C*07:02:01 (p-value=0.03) alleles, known to be involved in activating the immune response. Non-classical *HLA* subjects also presented peculiar clinical features compared to classical HLA subjects, such as multiple diabetic antibodies and the absence of other autoimmune diseases (i.e., coeliac disease and thyroiditis). We also observed that subjects with early onset had a higher frequency of DQ2/DQ8 genotype than late-onset individuals. Moreover, subjects with late-onset had a higher frequency of alleles HLA-B*27 (p-value=0.003), HLA-C*01:02:01 (p-value=0.027) and C*02:02:02 (p-value=0.01), known to be associated with increased protection against viral infections.

**Discussion:**

This study reveals a broader involvement of the *HLA* locus in the development and onset of type 1 diabetes, providing insights into new possible disease prevention and management strategies.

## 1 Introduction

Type 1 diabetes (T1D) is an autoimmune disease characterized by insulin deficiency due to chronic immune-mediated progressive destruction of pancreatic beta cells through the interaction between T lymphocytes and autoantigens (1, 2).

Potential triggers of pancreatic islet autoimmunity are viral infections, toxins, and dietary factors that act on a genetically susceptible background to T1D development (3–5). Over 60 loci across the genome were associated with T1D susceptibility (6, 7). However, the primary susceptibility locus is mapped to the *Human Leukocyte Antigen* (*HLA*) class II gene, placed on the short arm of chromosome 6, 6p21.31 (6, 8).

As the *HLA* system is highly polymorphic, numerous *HLA* alleles affect the peptides pool, which is recognized as initiating an immune reaction (9).

The strong linkage disequilibrium (LD) in the *HLA* region leads to the formation of haplotypes consisting of the combination of definite allelic variants. Specific *HLA* haplotypes are strongly associated with T1D (10) and those associated with the highest risk are HLA-DRB1*04-DQA1*03:01-DQB1*03:02 (or DR4-DQ8) and HLA-DRB1*03:01-DQA1*05:01-DQB1*02:01 (or DR3-DQ2) (11).

On the other hand, other *HLA* class II haplotypes, such as the DQB1*06:02-DRB1*15:01-DQA1*01:02, which is carried by 20% of the general population compared to 1% of T1D subjects, turn out to have a protective effect against the development of T1D (12).

*HLA* class II genes do not represent all the observed *HLA*-related inheritance of T1D (9) and some studies had also revealed the role of *HLA* class I genes (*HLA-A*, *HLA-B*, and *HLA-C*). The *HLA* class I molecules are involved in immune response, influencing beta cell destruction, as confirmed by the observation of overexpression of *HLA* class I molecules on islet cells of deceased T1D subjects (13). For example, HLA-A*24 alleles have been associated with rapid and complete destruction of beta cell function in T1D subjects (14); HLA-B*39:06 allele is known to be the most T1D predisposing *HLA* class I allele. At the same time, the HLA-B*57:01 has a protective effect (15). Conditional analyses, performed to exclude the effect of LD across the *HLA* region, also revealed an influence of the *HLA-DP* alleles on T1D susceptibility. In particular, the study by Varney et al. showed a predisposing effect of the DPA1*01:03 allele in association with the DPB1*03:01, whereas DPA1*01:03 has a protective role when associated with DPB1*04:02 (16).

Th e *HLA* genes also influence the age at onset of T1D. The high-risk genotype HLA-DRB1*03-DQA1*0501-DQB1*0201/DRB1*0401-DQA1*0301-DQB1*0302 was strongly associated with the onset of diabetes before the age of 5 years and that the frequency of this genotype in T1D subjects decreased with increasing age at onset (17).

Similarly, an association between *HLA* class I genes and the age at onset of diabetes was observed. For example, HLA-A*24:02, related to the total beta cell destruction, has been associated with an earlier onset of T1D (18). At the same time, diabetic subjects carrying HLA-B*39:06 had an average age at onset 3.7 years lower than subjects without this allele. The same trend was observed for T1D subjects carrying HLA-C*07:02; in contrast, we observed the opposite for carriers of the HLA-B*44:03, who had an age at onset approximately 3.5 years higher than people with diabetes without this allele (19).

Despite this growing body of work, to date, *HLA* screening in T1D individuals is limited to the determination of DR3 and DR4 haplotypes. Moreover, the previously mentioned studies reporting an involvement of *HLA* genes in the development of T1D have been conducted on a limited number of subjects and on a limited set of *HLA* genes.

However*, HLA* typing is fundamental for studying the risk of T1D and the pathogenesis of the disease, especially with a view to prevention and intervention in high-risk subjects, such as individuals with family history. Moreover, determining the genetic component underlying the early onset of T1D can lead to early intervention and avert greater severity of the disease. Indeed, numerous studies indicate that an early onset of T1D is associated with a poor prognosis of diabetes and an elevated risk of developing diabetes-related diseases (20–23).

Therefore, in this study, DNA samples from 115 children and young adults with T1D were typed for *HLA-A*, *-B*, *-C*, *-DRB1*, *-DRB3*/*4*/*5*, *-DQA1*, *-DQB1*, *-DPA1* and *-DPB1* by NGS (Next Generation Sequencing), with the aim of investigating the role of these genes on T1D risk and age at onset.

## 2 Materials and methods

### 2.1 Study participants

In this observational study, we included 115 T1D European subjects referred to the Endocrinologic Unit of IRCCS Burlo Garofolo of Trieste (Italy). T1D subjects were collected from June 2016 to August 2023 according to the following inclusion criteria: diagnosis of type 1 diabetes for at least one year and age between 6 and 21 years.

For all participants, demographic and anthropometric information (age, sex, height, weight, Body Mass Index (BMI), puberty) at the time of recruitment were collected. BMI standard deviation scores were calculated according to WHO reference charts (24) using the Growth Calculator 4 software (http://www.weboriented.it/gh4/), as recommended by the Italian Society for Pediatric Endocrinology and Diabetology (ISPED).

Puberty was defined as the presence of at least breast budding in girls [B2] and a testicular volume of 4 ml in boys [G2] (25).

Furthermore, we collected data relating to the T1D onset, such as the HbA1c value, the presence or absence of ketoacidosis (DKA) [calculated as a venous pH of <7.3 and/or a bicarbonate (HCO3) level of <15 mmol/L (26)], age at onset and the presence of antibodies: Insulin-directed antibodies (IAA), antibodies directed against tyrosine phosphatase (IA2), antibodies directed against the cytoplasm of pancreatic islet cells (ICA), antibodies directed against glutamic acid decarboxylase (GAD) and zinc transporter 8 autoantibodies (ZnT8A) (27).

We gathered additional clinical data, such as the previous year’s HbA1c of and the presence of diseases concomitant to diabetes (e.g., coeliac and thyroid disease).

All the enrolled participants or their parents, for participants aged <18 years, gave written informed consent. The ethics committee approved the study (CEUR-2018-Em-323-Burlo).

### 2.2 DNA extraction and *HLA* typing by next generation sequencing

DNA was extracted from saliva using the EZ1 DNA investigator kit (Qiagen, Milan, Italy) following the manufacturer’s protocols.

*HLA* typing was performed employing the MIA FORA NGS MFlex HLA typing kit (Immucor, Inc., GA, USA), which uses long-range PCR to capture the clinically relevant class I and II *HLA* genes. The kit includes each class I genes, *HLA-A*, *-B*, *-C*, and the class II genes, *HLA-DRB*, *-DQA1, -DQB1*, *-DPA1* and *-DPB1*.

Sample and sequencing library preparation was performed following the manufacturer’s protocols. NGS sequencing libraries were prepared in sets of 24 samples and were sequenced using iSeq™ 100 Illumina^®^.

Candidate *HLA* alleles were computed and the final *HLA* typing was called and examined using the MIA FORA NGS EXPRESS analysis software (Immucor, Inc., GA, USA).

Using three orthogonal algorithms, including independent mapping and *de novo* assembly strategies, a probability score was calculated, genotype candidates were ranked, and consensus sequences were created for individual alleles.

Due to the lack of coverage in the intron region, ambiguities in the fourth field could not be ruled out so *HLA* typings were assigned to the third field.

### 2.3 Statistical analysis

Demographic, anthropometric and clinical data were reported in contingency tables.

Continuous variables were indicated by mean and standard deviation (mean ± sd), and binary traits by percentages (%).

T1D subjects were grouped into classical *HLA* (CH) and non-classical *HLA* (NCH) according to the presence or absence of classical *HLA* haplotypes predisposing to diabetes.

According to percentile distribution of age at onset, T1D subjects were stratified into three groups: early-onset (EO) (age at onset <5 years old); intermediate-onset (IO) (age at onset ≥5<10 years old); late-onset (LO) (age at onset ≥10 old).

Poor Glycemic Control (PGC) [HbA1c > 7% (53 mmol/mol)] was defined using the mean HbA1c values of the previous year (28).

To test normality, we calculated skewness and kurtosis. We applied for binary variables the Fisher’s exact test to analyzed anthropometric and clinical data among the stratified T1D subjects. Instead, to compare continuous variables between CH and NCH T1D subjects, when the variable was normally or non-normally distributed, we used t-test or Mann-Whitney test, respectively.

Whereas, to compare continuous variables between EO, IO and LO T1D subjects, the ANOVA test for normal data and the Kruskall-Wallis test for non-normal data were used.

Fisher’s exact tests analyzed differences in *HLA* alleles and haplotypes between CH and NCH, and between EO, IO, and LO.

We set statistical significance at a p-value ≤ 0.05. All statistical analyses were performed with R software (www.r-project.org).

## 3 Results

### 3.1 HLA typing

We recruited 115 T1D children and young adults and performed *HLA* typing.

**Figure 1:**
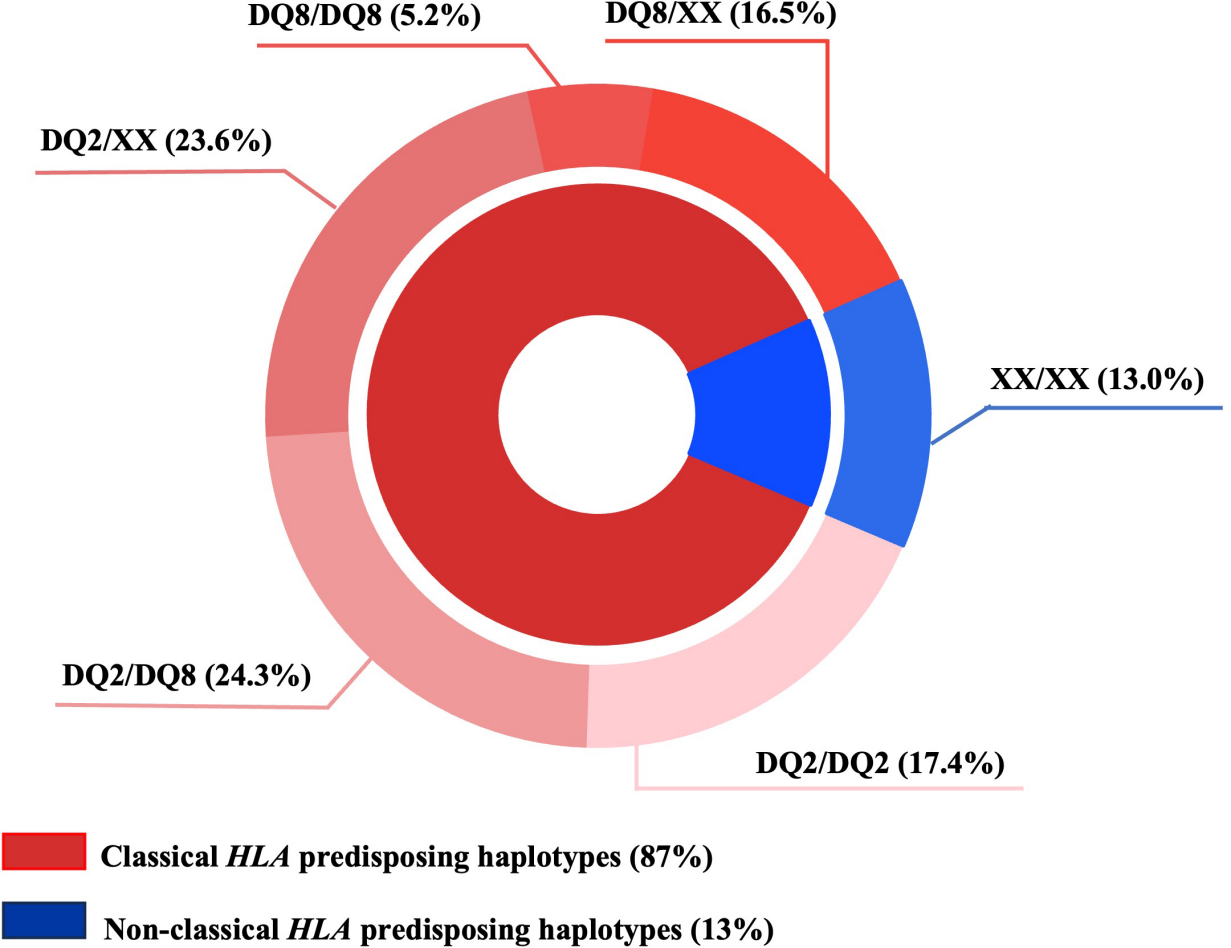
*HLA* haplotypes distribution in T1D subjects. XX≠DQ2 and/or DQ8.

We observed that in 87% of T1D subjects classical *HLA* predisposing haplotypes were present, represented mainly by the DQ2/DQ8 (24.3%) and the DQ2/XX (23.6%) genotypes (XX indicates a haplotype different from DQ2 and/or DQ8), as shown in the pie chart (**Figure 1**). Interestingly, *HLA* typing also showed that 13% of T1D subjects had non-classical *HLA* haplotypes predisposing to diabetes.

**Figure 2:**
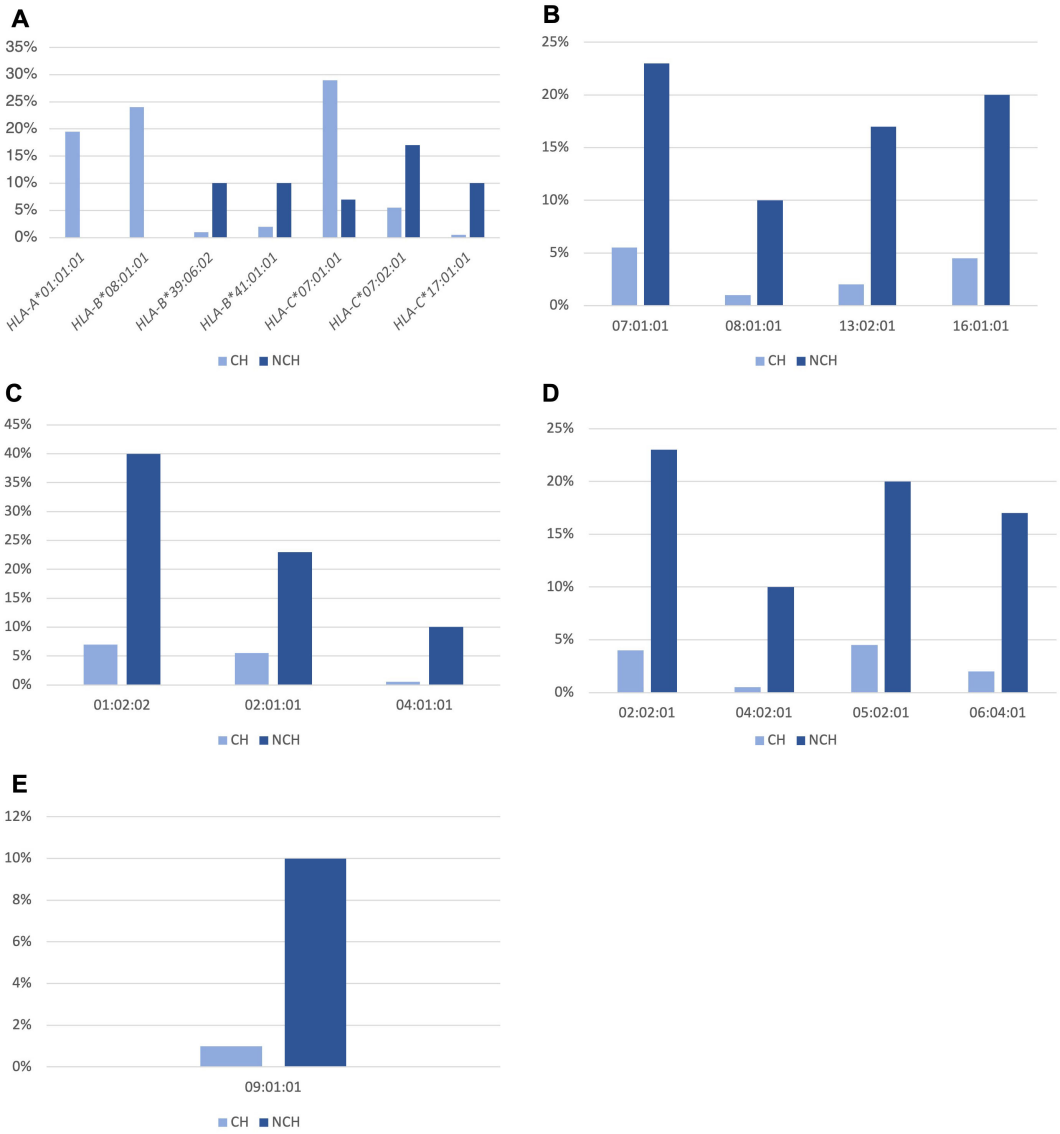
Statistically different allele frequencies in the *HLA-A, -B* and -C genes of class I **(A) **and in the *HLA-DRB1* **(B)**, *-DQA1* **(C)**, *-DQB1* **(D)** and -*DPB1* **(E) **genes of class II, in T1D subjects stratified according to the presence of *HLA* haplotypes predisposing to T1D, in subjects with classical *HLA* (CH) and non-classical *HLA* (NCH).

Analysis of the allele frequencies of *HLA* class I and II genes in CH and NCH T1D subjects showed statistically different allele frequencies between T1D CH and NCH groups (**Figure 2**). **Supplementary Table 1** reports all *HLA* alleles identified in T1D subjects. In particular, for the *HLA* class I alleles, we found that HLA-A*01:01:01 (19.5% *vs.* 0%, p-value = 0.002), HLA-B*08:01:01 (24% *vs.* 0%, p-value = 0.0003) and HLA-C*07:01:01 (29% *vs.* 7%, p-value = 0.01) were more frequent in CH T1D that in NCH T1D subjects (**Figure 2A**). Instead, *HLA* class I alleles more frequent in NCH T1D than CH T1D subjects were HLA-B*39:06:02 (10% *vs.* 1%, p-value = 0.01), HLA-B*41:01:01 (10% *vs.* 2%, p-value = 0.04), HLA-C*07:02:01 (17% *vs.* 3.5%, p-value = 0.03), and HLA-C*17:01:01 (10% *vs.* 0.5%, p-value = 0.007) (**Figure 2A**).

For *HLA* class II alleles, those which were part of the *HLA DQ2* and *DQ8* haplotypes of predisposition to T1D were more frequent in CH T1D subjects than in NCH subjects, i.e., HLA-DRB1*03:01:01 (44.5% *vs.* 0%, p-value < 0.0001), DRB1*04:01:01 (12.5% *vs.* 0%, p-value = 0.03); HLA-DQA1*03:01:01 (28% *vs.* 7%, p-value = 0.01), HLA-DQA1*05:01:01 (45.5% *vs.* 0%, p-value < 0.0001); HLA-DQB1*02:01:01 (45.5% *vs.* 7%, p-value < 0.0001) and HLA-DQB1*03:02:01 (30% *vs.* 3%, p-value = 0.0005).

In NCH T1D subjects, the *HLA* class II alleles significantly more frequent concerning CH T1D subjects were: HLA-DRB1*07:01:01 (23% *vs.* 5.5%, p-value = 0.002), HLA-DRB1*08:01:01 (10% *vs.* 1%, p-value = 0.01), HLA-DRB1*13:02:01 (17% *vs.* 2%, p-value = 0.009) and HLA-DRB1*16:01:01 (20% *vs.* 4.5%, p-value = 0.02) (**Figure 2B**); HLA-DQA1*01:02:02 (40% *vs.* 7%, p-value = 0.001), HLA-DQA1*02:01:01 (23% *vs.* 5.5%, p-value = 0.002) and HLA-DQA1*04:01:01 (10% *vs.* 0.5%, p-value = 0.007) (**Figure 2C**); HLA-DQB1*02:02:01 (23% *vs.* 4%, p-value = 0.002), HLA-DQB1*04:02:01 (10% *vs.* 0.5%, p-value = 0.007), HLA-DQB1*05:02:01 (20% *vs.* 4.5%, p-value = 0.02) and HLA-DQB1*06:04:01 (17% *vs.* 2%, p-value = 0.009) (**Figure 2D**); HLA-DPB1*09:01:01 (10% *vs.* 1%, p-value = 0.01) (**Figure 2E**).

### 3.2 Characteristics of T1D subjects according to the presence of classical and non-classical HLA haplotypes

In **Table 1**, we reported the characteristics of T1D subjects stratified according to the presence of classical and non-classical *HLA* haplotypes predisposing to T1D.

**Table 1:** Characteristics of the T1D subjects stratified according to *HLA* haplotypes predisposing to T1D in classical *HLA* (CH) and non-classical *HLA* (NCH) subjects.

	All (*n*=115)	CH (*n*=100)	NCH (*n*=15)	p-value
Sex, Female	54%	54%	53%	1
Age (years)	15.6 ± 3.7	15.7 ± 3.5	14.7 ± 4.7	0.59
Disease duration (years)	8.1 ± 4.2	8.4 ± 4.2	6.1 ± 3.5	**0.04**
Age at onset (years)	7.5 ± 3.9	7.4 ± 3.7	8.6 ± 5.3	0.42
HbA1c at onset %	11.0 ± 2.4	10.9 ± 2.4	11.5 ± 2.3	0.45
HbA1c %	7.6 ± 1.2	7.7 ± 1.3	7.3 ± 0.7	0.53
PGC (HbA1c > 7%)	65%	66%	60%	0.77
DKA at onset	28%	26%	38.5%	0.34
SDS-BMI	0.24 ± 1.1	0.30 ± 1.0	-0.19 ± 1.3	0.22
Pubertal Status	89%	90%	80%	0.37
T1D Ab				
IAA	33%	32%	40%	0.72
IA2	59%	57%	70%	0.51
ICA	20%	20.5%	20%	1
GAD	79%	76.5%	100%	0.11
ZnT8A	20%	19%	30%	0.42
Ab >1	70.5%	66%	100%	**0.02**
Coeliac disease	14%	16.5%	0%	0.12
Autoimmune thyroiditis	18%	21%	0%	0.07
CSII	65%	66%	60%	0.77

Data are shown as mean ± SD or %.

PGC, Poor glycemic control; DKA, Diabetic ketoacidosis; SDS-BMI, Standardized Body Mass Index; Ab, Antibody; CSII, Continuous subcutaneous insulin infusion.

In bold are indicated p values <0.05.

Differences among T1D subjects were computed by the Fisher exact test, t-test and by Mann-Whitney U test, as appropriate.

Overall, 54% of the 115 T1D subjects included in the study were female, the mean age of the sample was 15.6 ± 3.7 years, 28% presented DKA at onset, and 65% had a PGC.

Despite the limited number of NCH subjects, comparing CH and NCH subjects, we found that NCH T1D subjects had a shorter disease duration than the CH T1D ones (6.1 *vs.* 8.4 years old, p-value = 0.03) and that all NCH subjects had more than one antibody compared to 66% of CH T1D subjects (p-value = 0.02).

Furthermore, although differences did not reach statistical significance, higher age at onset was observed in NCH subjects compared to CH T1D subjects (8.5 *vs.* 7.4 years), as well as a more elevated percentage of DKA at onset in NCH compared to CH (38.5% *vs.* 26%). Finally, none of the NCH T1D subjects showed coeliac disease or autoimmune thyroid disease, compared to CH T1D subjects (16.5% and 21%, respectively).

### 3.3 Association between HLA and age at onset

In the present work, we stratified subjects according to their age at onset of T1D and studied its association with HLA genotypes.

In our study population, the age at onset was <5 years old (early-onset, EO) in 31% of participants, between 5 and 10 years old (intermediate-onset, IO) in 46%, and over 10 years old (late-onset, LO) in the remaining 23%. In EO, IO and LO groups, respectively 53%, 45% and 73% were females, while the mean age in the three groups was 13.9 ± 4.1, 15.8 ± 3.5 and 17.5 ± 2.5. Anthropometric and clinical data are reported in **Supplementary Table 2**.

**Table 2** indicates the genotype distribution according to age at onset. The most frequent HLA genotype was DQ2/DQ8, followed by the DQ2/XX genotype, except for LO T1D subjects, for whom the most frequent genotype was DQ2/XX, followed by DQ8/XX.

**Table 2:** Genotype distribution in T1D subjects stratified according to age at onset in early-onset (EO, <5 years old), intermediate-onset (IO, ≥5<10 years old) and late-onset (LO, ≥10 years old).

*HLA* class II genotypes	EO (*n*=36)	IO (*n*=53)	LO (*n*=26)	p-value
DQ2/XX	22%	23%	27%	0.69
DQ2/DQ2	19%	17%	15%	0.67
DQ8/XX	14%	17%	19%	0.57
DQ8/DQ8	3%	6%	8%	0.39
DQ2/DQ8	31%	26%	12%	0.10
XX/XX	11%	11%	19%	0.38

XX≠DQ2 and/or DQ8.

Differences among T1D subjects were computed by the Fisher exact test.

**Figure 3:**
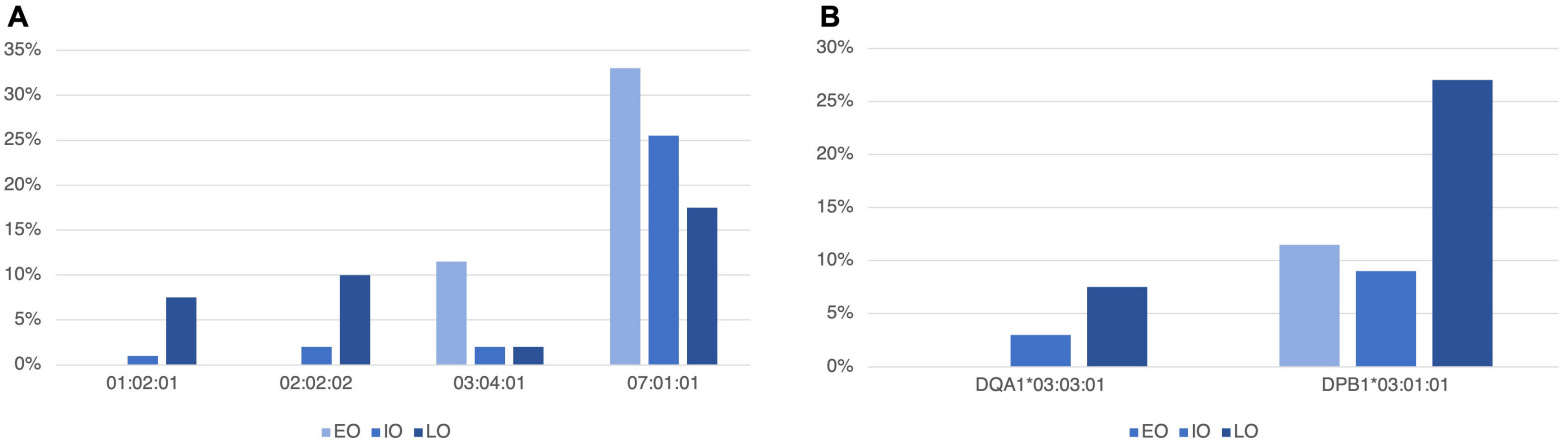
Statistically different allele frequencies in the *HLA-C* gene of class I **(A)** and in the *HLA-DQA1* and *-DPB1* **(B)** class II genes, in T1D subjects, stratified according to age at onset in early-onset (EO, <5 years old), intermediate-onset (IO, ≥5<10 years old) and late-onset (LO, ≥10 years old).

In T1D subjects, stratified according to age at onset in EO, IO and LO, no significant differences emerged in genotypes distribution. Instead regarding the frequency of *HLA* alleles, **Figure 3** reports significant differences in the three groups. In particular, for the HLA-C locus, HLA-C*01:02:01 and *02:02:02 were more frequent in LO than in EO (7.5% *vs.* 0%, p-value = 0.027, 10% *vs.* 0%, p-value = 0.01, respectively), and in IO (7.5% *vs.* 1% p-value = 0.038, 10% *vs.* 2%, p-value = 0.036, respectively), while HLA-C*03:04:01 was more frequent in EO (11.5%) compared to IO (2%) and LO (2%) but only the comparison between EO and IO was statistically significant (p-value = 0.01).

HLA-C*07:01:01 was also more frequent in EO (33%) compared to IO (25.5%) and LO (17.5%). In this case, only the comparison between EO and LO was statistically significant (p-value = 0.04).

Concerning *HLA* class II alleles, we found that HLA-DQA1*03:03:01 was more frequent in LO T1D subjects than in EO, in which this allele was absent (7.5% *vs.* 0%, p-value = 0.027). HLA-DPB1*03:01:01 was also more frequent in LO (27%) compared to EO (11.5%) and IO (9%) (p-value = 0.029 and p-value = 0.016, respectively).

**Supplementary Table 3** reports all *HLA* class I and II allele frequencies in EO, IO and LO. A particular mention should be made about HLA-B*27 alleles (*27:02:01*, *27:05:02* and *27:07:01*), which were not detected in EO subjects, whereas they were present in 2% of IO and 23% of LO, with significant differences in the comparison between EO and LO (p-value = 0.003) and between IO and LO (p-value = 0.004).

## 4 Discussion

Our findings suggest that haplotypes strongly associated with the development of T1D are not the only *HLA* genetic component involved in the risk of diabetes and in the age at onset of the disease.

In our cohort, DQ2/DQ8 was the most frequent genotype, in agreement with previous literature data (29, 30). However, in our study *HLA* high-resolution typing proved that 13% of T1D subjects did not have the classical *HLA* haplotypes predisposing to the disease. In agreement with our results, approximately the same frequency of T1D subjects without classic *HLA* predisposition alleles was already found in Polish T1D children and adolescents (31), confirming that *HLA* class II alleles do not represent the entire genetic component of predisposition to T1D.

In particular, in the present study, analysis of the genetic characteristics of T1D subjects without the classical *HLA* predisposing haplotypes showed that the *HLA* class II alleles significantly more frequent in NCH T1D subjects were part of four different haplotypes (HLA-DRB1*07:01:01-DQA1*02:01:01-DQB1*02:02:01; HLA-DRB1*08:01:01-DQA1*04:01:01-DQB1*04:02:01; HLA-DRB1*13:02:01-DQA1*01:02:02-DQB1*06:04:01; and HLA-DRB1*16:01:01-DQA1*01:02:02-DQB1*05:02:01), previously reported to have a neutral or, even, a protective role towards the T1D development risk (29, 32, 33).

These data suggested that NCH T1D subjects probably had other genetic risk factors, which may reside in the *HLA* class I region. The role of *HLA* class I genes on the development of T1D had indeed already been assessed, as the encoded molecules bind and present antigens to CD8^+^ T cells, both by aiding the selection of the T cell repertoire and by initiating antigen-specific cytotoxicity (30).

In our study, one of the more frequent *HLA* class I alleles in NCH compared to CH T1D subjects was HLA-B*39:06:02. This allele was already known to be the most predisposing *HLA* class I allele to T1D (15, 34). By developing an HLA-B*39:06 transgenic NOD mouse model, Schloss et al. (35) demonstrated that this allele independently mediates the development of CD8^+^ T cells that conferred susceptibility to T1D. Some studies reported that HLA-B*39:06 could increase the risk of diabetes in combination with some low-risk haplotypes, containing DRB1*08:01, DRB1*01:01 or DRB1*16:01 alleles (19, 36). The odds ratio values obtained from combination of HLA-B*39:06 with these alleles indicated a significantly raised risk, comparable to that of the DR3/DR4 genotype (36). In our sample, HLA-B*39:06 was indeed found in combination with these alleles, confirming the hypothesis of its involvement in the development of T1D, even in the absence of high-risk haplotypes.

Another *HLA* class I allele that we have frequently found in NCH T1D subjects was HLA-C*07:02:01. This allele was positively correlated with increased expression of the T cell receptor complex variable region (TCR V) gene, stimulating high immunological activity (37), as suggested by its known association with T1D (34) and psoriasis (37). Some authors hypothesized that the effect of this allele on the development of T1D depended on LD with HLA-B*39:06. However, its strong association with the early onset of diabetes, independently from *HLA*-B*39:06, supported an autonomous effect on predisposition to T1D (19). In our cohort, we did not find a LD between these two alleles; furthermore, the HLA-C*07:02:01 was always in combination with protective or neutral DR-DQ haplotypes, confirming its autonomous effect on the T1D risk.

Interestingly, the present work suggests that diabetic individuals without the classical high-risk haplotypes present peculiar clinical characteristics. For example, we found that all NCH subjects showed more than one diabetes antibody compared to 66% of the CH group. Our results did not confirm previous studies that reported the role of *HLA-DR3* and *HLA-DR4* alleles in producing T1D antibodies (38–41). However, the role of other genes has also been proposed (40–42), and future studies should be conducted to investigate their involvement in antibody development better.

In our work, other clinical characteristics were peculiar to NCH T1D subjects, even if the comparison with CH did not result statistically significant, possibly due to the small sample size of our study. For example, we observed that 38% of NCH T1D subjects presented DKA at disease onset, compared to 26% of CH T1D subjects, in agreement with previous studies that had already shown a protective effect of high-risk *HLA* alleles on the development of DKA (43, 44). Moreover, NCH T1D subjects did not present other autoimmune pathologies, such as coeliac disease and autoimmune thyroid diseases. This observation highlighted the usefulness of carrying out extended *HLA* typing in helping to identify distinct clinical characteristics and set or avoid specific controls. For example, implementing genetic testing could reduce the number of patients requiring systematic immunological screening.

Finally, the age at onset of the disease in the NCH T1D group was slightly older than in the CH T1D subjects, confirming previous studies on the role of *HLA* genes in this regard (18, 19, 45).

Infact, investigating the association of *HLA* genes according to the age at onset, we also observed that T1D subjects with early onset had a higher percentage of DQ2/DQ8 genotype than T1D subjects with late onset. This result suggested that the role of this genotype is not only to increase the risk of developing T1D but also to induce its development at an early age. Although it was difficult to determine whether this high-risk genotype alone was enough to confer a greater risk of developing diabetes early, we showed that the DQ2/DQ8 genotype was associated with an early age at onset (6.37 age at onset) and that the age at onset increased in the absence of DQ2 and in the presence of DQ8 (DQ2/DQ2 = 7.07 age at onset; DQ2/XX = 7.8 age at onset; DQ8/XX = 8.1 age at onset; DQ8/DQ8 = 8.9; XX/XX = 8.6). Interestingly, when we evaluated the mean age at onset associated with both DQ2/DQ8 genotype and HLA-C*03:04:01, that were significantly more frequent in the early onset subjects, we observed that age at onset lowered to 3.9 years. This observation implied that other *HLA* genetic factors might be involved in early onset besides DQ2 and DQ8.

On the other hand, in the group of late onset T1D subjects, the significantly more frequent *HLA* alleles were HLA-B*27 alleles, HLA-C*01:02:01 and C*02:02:02, all known to be associated with increased protection against viral infections (46–49). Since the peak of early onset observed around the age of 5 was connected to the numerous infections in the early school years (50), protection from viral infections given by these alleles could prevent the early development of the disease. The presence of the alleles mentioned above delayed the age at onset by about 5 years compared to T1D subjects not carrying these alleles (HLA-B*27+, average age at onset 12.48, HLA-B*27- average age at onset 7.21; HLA-C*01:02:01+, average age at onset 11.65, HLA-C*01:02:01-, average age at onset 7.34; HLA-C*02:02:02+, average age at onset 12.14, HLA-C*02:02:02-, average age at onset 7.23), independently from classical *HLA* risk haplotypes.

Interestingly, some of these *HLA* alleles were also strongly associated with the development of other diseases, such as seronegative arthritis (HLA-B*27) (51) and psoriasis (HLA-C*01) (52). It means that these subjects, protected from the early development of diabetes, could be more susceptible to the development of other autoimmune diseases. In our study, T1D subjects did not manifest any *HLA*-related diseases (excluding coeliac and autoimmune thyroid diseases). However, our outcomes suggested that this risk should be considered when screening of diabetic subjects.

Overall, the findings of this study highlight important implications for future clinical practice.

First, our study supports the use of *HLA* typing by NGS as an accurate and feasible method for *HLA-*related disease association testing (including T1D), allowing to overcome the detection limitations of low-resolution traditional methods commonly used in clinical practice [i.e., Sequence-Specific Oligonucleotide (SSO) and Sequence-Specific Primer (SSP)] (53). Moreover, considering that the NGS costs continue to decline and are currently similar to or lower than the SSO method, *HLA* typing by NGS may also become a cost-effective approach, especially in large volume laboratories, thanks to the possibility of analyzing many patients within a single run.

Furthermore, our results underline the possible relevance of *HLA* extended typing from a prevention perspective. The purpose of screening for T1D is to identify early the risk of developing the disease to prevent a severe onset of diabetes with the presence of DKA, causing both short- and long-term complications. Current T1D screening, in addition to analyzing the presence of antibodies, only assesses the presence of the primary genetic risk factors, DR3 and DR4. In a pediatric population screening context, *HLA* extended typing would allow the identification of new diabetes predisposition alleles whose identification requires a sample size to counteract the excessive allelic variability of the *HLA* locus. Moreover, considering their genetic heterogeneity would allow a more accurate genetic risk assessment of different populations. So, if NGS becomes widely used, it could provide a valuable resource for future screening and population studies.

The results of our study show that *HLA* is associated with clinical characteristics, including age at onset, also emphasize that NGS-based *HLA* analyses may be relevant for defining clinical phenotypes and predicting the disease course. Moreover, since the *HLA* region represents the major genetic component for the development of numerous autoimmune diseases, such as coeliac disease, psoriasis, rheumatoid arthritis, and thyroiditis, often concomitant in diabetic subjects, NGS *HLA* extended typing could also prove useful in the management of T1D subjects (54).

Overall, although low-resolution *HLA* typing continues to be used in clinical practice, an NGS-based method should be considered in the future since more precise assignments of *HLA* alleles/haplotypes could help to elucidate the exact role of *HLA* variation in the etiopathogenesis and progression of T1D, which may result in improved patient care, decreased additional testing, and then reduced costs.

## 5 Conclusion

The study’s results indicate that the *HLA* locus’s involvement in the development of T1D and the onset time occurs on a broader spectrum and does not only involve the classical *HLA* genotypes known to play a predisposing role in diabetes.

However, despite high-resolution genotyping, the extreme allelic variability, in conjunction with the limited number of subjects analyzed, makes these data preliminary. Therefore, it is essential to increase the number of T1D subjects studied to better understand the role of the *HLA* genes in type 1 diabetes. Moreover, given the small sample size, some associations may have been missed. Furthermore, control group of healthy subjects are not considered in this study.

Overall, despite these limitations, the results of our study suggest that an in-depth understanding of the *HLA* genetic variations associated with T1D and, in particular, with age at onset, may increase our knowledge of the mechanisms underlying the development of the disease and can also be used in disease prevention and in patient management.

## Data Availability

The original Fastq files obtained from HLA typing are publicly available at https://www.ebi.ac.uk/ena/browser/home with access ID: PRJEB76550. Other information may be available from the corresponding author upon reasonable request.
