# Supplementary material for: Next-generation sequencing reveals additional HLA class I and class II alleles associated with type 1 diabetes and age at onset
Source: Front Immunol. 2024 Aug 9;15:1427349. doi: 10.3389/fimmu.2024.1427349 (PMC11341356; doi:10.3389/fimmu.2024.1427349)
Supplement: Supplementary file 1 [file Table_1.docx]

**Supplementary Table 1**: *HLA* class I and class II allele frequencies in T1D subjects stratified according to *HLA* haplotypes predisposing to T1D in classical *HLA* (CH) and non-classical *HLA* (NCH) subjects.

| *HLA* allele | All  (2n=230) | CH  (2n=200) | NCH  (2n=30) |
| --- | --- | --- | --- |
| ***HLA class I*** | | | |
| ***HLA-A**** |  |  |  |
| 01:01:01 | 17% | 19.5% | - |
| 02:01:01 | 22% | 20% | 33% |
| 02:02:01 | 1.5% | 1% | 3.5% |
| 02:05:01 | 5% | 4.5% | 6.5% |
| 02:11:01 | 0.5% | 0.5% | - |
| 03:01:01 | 6.5% | 6.5% | 6.5% |
| 03:02:01 | 3% | 3.5% | - |
| 11:01:01 | 4.5% | 5% | 3.5% |
| 23:01:01 | 1.5% | 2% | - |
| 24:02:01 | 17% | 16.5% | 20% |
| 25:01:01 | 1.5% | 1.5% | 3.5% |
| 26:01:01 | 1.5% | 1.5% | 3.5% |
| 29:01:01 | 1% | 1% | - |
| 29:02:01 | 2% | 1% | 6.5% |
| 30:02:01 | 3% | 3.5% | - |
| 30:04:01 | 1.5% | 1.5% | - |
| 31:01:02 | 1.5% | 2% | - |
| 32:01:01 | 3% | 3% | 3.5% |
| 32:02 | 0.5% | 0.5% | - |
| 33:01:01 | 1% | 1% | - |
| 68:01:01 | 4% | 4% | 6.5% |
| 68:24 | 0.5% | 0.5% | - |
| 68:25 | 0.5% | - | 3.5% |
| ***HLA-B**** |  |  |  |
| 07:02:01 | 3.5% | 2.5% | 10% |
| 07:05:01 | 1% | 0.5% | 3% |
| 07:06:02 | 1% | 1% | - |
| 08:01:01 | 21% | 24% | - |
| 13:02:01 | 1.5% | 1.5% | 3% |
| 14:02:01 | 1.5% | 2% | - |
| 15:01:01 | 5.5% | 6% | 4% |
| 15:17:01 | 1.5% | 1% | 7% |
| 15:220:01 | 0.5% | 0.5% | - |
| 18:01:01 | 9.5% | 10% | 7% |
| 18:03:01 | 0.5% | 0.5% | - |
| 27:02:01 | 0.5% | 0.5% | - |
| 27:05:02 | 2% | 2.5% | - |
| 27:07:01 | 0.5% | 0.5% | - |
| 35:01:01 | 4.5% | 5% | 3% |
| 35:02:01 | 0.5% | 0.5% | - |
| 35:03:01 | 3% | 3% | - |
| 35:08:01 | 1.5% | 2% | - |
| 37:01:01 | 1% | 1% | - |
| 38:01:01 | 2% | 1.5% | 7% |
| 39:01:01 | 3.5% | 2.5% | 10% |
| 39:06:02 | 2% | 1% | 10% |
| 40:01:02 | 0.5% | 0.5% | - |
| 40:02:01 | 1.5% | 2% | - |
| 41:01:01 | 3% | 2% | 10% |
| 44:02:01 | 1.5% | 2% | - |
| 44:03:01 | 3% | 2.5% | 3% |
| 45:01:01 | 0.5% | 0.5% | - |
| 49:01:01 | 3% | 3% | - |
| 50:01:01 | 5.5% | 5.5% | 7% |
| 51:01:01 | 6% | 6% | 7% |
| 55:01:01 | 2% | 2% | 3% |
| 57:01:01 | 1.5% | 1.5% | - |
| 57:03:01 | 0.5% | - | 3% |
| 58:01:01 | 3% | 2.5% | 3% |
| 73:01:01 | 0.5% | 0.5% | - |
| ***HLA-C**** |  |  |  |
| 01:02:01 | 2.5% | 2% | 4% |
| 02:02:02 | 3% | 3.5% | - |
| 03:02:02 | 1% | 1% | - |
| 03:03:01 | 4% | 4% | 4% |
| 03:04:01 | 5% | 5.5% | - |
| 04:01:01 | 9.5% | 9.5% | 10% |
| 05:01:01 | 6% | 7% | - |
| 06:02:01 | 10% | 10% | 10% |
| 07:01:01 | 26% | 29% | 7% |
| 07:02:01 | 7% | 5.5% | 17% |
| 07:18:01 | 1.5% | 1.5% | 3% |
| 08:02:01 | 1.5% | 2% | - |
| 12:03:01 | 10% | 8% | 23% |
| 14:02:01 | 1.5% | 2% | **-** |
| 14:03:01 | 1% | 1% | - |
| 15:02:01 | 3.5% | 3.5% | 3% |
| 15:05:02 | 1.5% | 1.5% | 3% |
| 16:01:01 | 1.5% | 1.5% | 3% |
| 16:02:01 | 1% | 0.5% | 3% |
| 16:04:01 | 0.5% | 0.5% | - |
| 17:01:01 | 2% | 0.5% | 10% |
| 18:02:01 | 0.5% | 0.5% | - |
| ***HLA class II*** | | | |
| ***HLA-DRB1**** |  |  |  |
| 01:01:01 | 3.5% | 3% | 7% |
| 01:02:01 | 0.5% | - | 3% |
| 03:01:01 | 39% | 44.5% | **-** |
| 04:01:01 | 11% | 12.5% | **-** |
| 04:02:01 | 10% | 11.5% | - |
| 04:04:01 | 1.5% | 2.5% | - |
| 04:05:01 | 5% | 4.5% | 7% |
| 07:01:01 | 8% | 5.5% | 23% |
| 08:01:01 | 2% | 1% | 10% |
| 09:01:02 | 1% | 1% | - |
| 11:01:01 | 1.5% | 1.5% | 3% |
| 11:03:01 | 1.5% | 1.5% | - |
| 11:04:01 | 1% | 1% | - |
| 12:01:01 | 1.5% | 1% | 7% |
| 13:01:01 | 1.5% | 2% | - |
| 13:02:01 | 4% | 2% | 17% |
| 15:01:01 | 1% | 0.5% | 3% |
| 16:01:01 | 6.5% | 4.5% | 20% |
| ***HLA-DQA1**** |  |  |  |
| 01:01:01 | 4% | 3% | 10% |
| 01:02:02 | 11% | 7% | 40% |
| 01:03:01 | 1.5% | 2% | - |
| 01:63:01 | 0.5% | 0.5% | - |
| 02:01:01 | 8% | 5.5% | 23% |
| 03:01:01 | 25% | 28% | 7% |
| 03:02:01 | 1% | 1% | - |
| 03:03:01 | 3.5% | 3.5% | - |
| 04:01:01 | 2% | 0.5% | 10% |
| 05:01:01 | 39.5% | 45.5% | **-** |
| 05:05:01 | 4% | 3.5% | 10% |
| ***HLA-DQB1**** |  |  |  |
| 02:01:01 | 40% | 45.5% | 7% |
| 02:02:01 | 6.5% | 4% | 23% |
| 03:01:01 | 5% | 5% | 7% |
| 03:02:01 | 26.5% | 30% | 3% |
| 03:03:02 | 2% | 2.5% | - |
| 04:02:01 | 2% | 0.5% | 10% |
| 05:01:01 | 5% | 4% | 10% |
| 05:02:01 | 6.5% | 4.5% | 20% |
| 06:02:01 | 0.5% | - | 3% |
| 06:03:01 | 2% | 2% | - |
| 06:04:01 | 4% | 2% | 17% |
| ***HLA-DPA1**** |  |  |  |
| 01:03:01 | 80% | 80% | 80% |
| 01:04:01 | 1% | 1% | - |
| 02:01:02 | 18% | 18% | 17% |
| 02:02:02 | 1% | 1% | 3% |
| ***HLA-DPB1**** |  |  |  |
| 01:01:01 | 9.5% | 11% | - |
| 02:01:02 | 19% | 21% | 7% |
| 02:02:01 | 1% | 1% | - |
| 03:01:01 | 14% | 14.5% | 10% |
| 04:01:01 | 32% | 30.5% | 40% |
| 04:02:01 | 4% | 4% | 3% |
| 05:01:01 | 1% | 1% | 3% |
| 06:01:01 | 0.5% | 0.5% | - |
| 09:01:01 | 2% | 1% | 10% |
| 10:01:01 | 1% | 0.5% | 7% |
| 11:01:01 | 1% | 0.5% | 3% |
| 13:01:01 | 2% | 2% | - |
| 14:01:01 | 2% | 2.5% | - |
| 15:01:01 | 1% | 1.5% | - |
| 17:01:01 | 1% | 1% | - |
| 23:01:01 | 2% | 1% | 7% |
| 104:01:01 | 6% | 6% | 7% |
| 116:01 | 0.5% | 0.5% | - |
| 124:01:01 | 0.5% | - | 3% |

**Supplementary Table 2**: Anthropometric and clinical data in T1D subjects stratified according to age at onset in early-onset (EO, <5 years old), intermediate-onset (IO, ≥5<10 years old) and late-onset (LO, ≥10 years old)

Data are shown as mean±SD or %

PGC= Poor glycaemic control; DKA= Diabetic ketoacidosis; SDS-BMI= Standardized Body Mass Index; Ab= Antibody; CSII=Continuous subcutaneous insulin infusion.
In bold are indicated p values <0.05.
Differences among T1D subjects were computed by Fisher exact test, by the ANOVA test and by Kruskal-Wallis test, as appropiate.

|  | EO  (n=36) | IO  (n=53) | LO  (n=26) | p value |
| --- | --- | --- | --- | --- |
| Disease duration (years) | 10.9±4.5 | 7.9±3.2 | 4.5±2.2 | **<0.001** |
| HbA1c at onset % | 9.9±2.4 | 11.1±1.9 | 12.1±2.8 | 0.58 |
| HbA1c % | 7.9±1.5 | 7.5±1.0 | 7.5±1.1 | 0.33 |
| PGC (HbA1c >7%), | 78% | 60% | 58% | 0.08 |
| DKA at onset | 24% | 32% | 23% | 0.96 |
| SDS-BMI | 0.11±0.9 | 0.19±1.2 | 0.55±1.1 | 0.31 |
| Pubertal Status | 71% | 96% | 100% | **<0.001** |
| T1D Ab |  |  |  |  |
| IAA | 30% | 31% | 36% | 0.68 |
| IA2 | 61% | 61% | 45% | 0.31 |
| ICA | 9% | 22% | 27% | 0.12 |
| GAD | 74% | 72% | 86% | 0.34 |
| ZnT8A | 9% | 22% | 27% | 0.12 |
| Ab >1 | 56% | 75% | 68% | 0.40 |
| Coeliac disease | 28.5% | 10% | 4% | **0.004** |
| Autoimmune thyroiditis | 17% | 17% | 23% | 0.59 |
| CSII | 67% | 70% | 54% | 0.35 |

**Supplementary Table 3**: *HLA* class I and class II allele frequencies in T1D subjects stratified according to age at onset in early-onset (EO, <5 years old), intermediate-onset (IO, ≥5<10 years old) and late-onset (LO, ≥10 years old)

| *HLA* allele | EO  (2n=72) | IO  (2n=106) | LO  (2n=52) |
| --- | --- | --- | --- |
| ***HLA class I*** | | | |
| ***HLA-A**** |  |  |  |
| 01:01:01 | 14% | 20% | 15% |
| 02:01:01 | 24% | 17% | 29% |
| 02:02:01 | - | 2% | 2% |
| 02:05:01 | 3% | 7% | 2% |
| 02:11:01 | - | 1% | - |
| 03:01:01 | 5% | 7% | 5.5% |
| 03:02:01 | 3% | 3.5% | 2% |
| 11:01:01 | 8% | 2% | 5.5% |
| 23:01:01 | 3% | 2% | - |
| 24:02:01 | 21% | 17% | 11% |
| 25:01:01 | - | 3% | 2% |
| 26:01:01 | 1.5% | 2% | 2% |
| 29:01:01 | - | 1% | 2% |
| 29:02:01 | 1.5% | 2% | 2% |
| 30:02:01 | 1.5% | 3.5% | 4% |
| 30:04:01 | 1.5% | 2% | - |
| 31:01:02 | - | 2% | 4% |
| 32:01:01 | 5% | 1% | 4% |
| 32:02 | - | 1% | - |
| 33:01:01 | - | - | 4% |
| 68:01:01 | 8% | 2% | 4% |
| 68:24 | - | 1% | - |
| 68:25 | - | 1% | - |
| ***HLA-B**** |  |  |  |
| 07:02:01 | 4% | 5% | - |
| 07:05:01 | - | 1% | 2% |
| 07:06:02 | 1.5% | 1% | - |
| 08:01:01 | 26% | 19% | 17% |
| 13:02:01 | 1.5% | 3% | - |
| 14:02:01 | 1.5% | 1% | 4% |
| 15:01:01 | 8% | 5% | 2% |
| 15:17:01 | 4% | 1% | - |
| 15:220:01 | 1.5% | - | - |
| 18:01:01 | 11% | 6.5% | 13.5% |
| 18:03:01 | 1.5% | - | - |
| 27:02:01 | - | - | 2% |
| 27:05:02 | - | 1% | 7.5% |
| 27:07:01 | - | - | 2% |
| 35:01:01 | 1.5% | 5% | 7.5% |
| 35:02:01 | - | 1% | - |
| 35:03:01 | 4% | 2% | 2% |
| 35:08:01 | 1.5% | 2% | 2% |
| 37:01:01 | 1.5% | 1% | - |
| 38:01:01 | 1.5% | 2% | 4% |
| 39:01:01 | 4% | 2% | 5.5% |
| 39:06:02 | 4% | 2% | - |
| 40:01:02 | 1.5% | - | - |
| 40:02:01 | 2.5% | 2% | - |
| 41:01:01 | 1.5% | 5% | 2% |
| 44:02:01 | - | 2% | 4% |
| 44:03:01 | 1.5% | 3.5% | 2% |
| 45:01:01 | - | 1% | - |
| 49:01:01 | 3% | 3.5% | - |
| 50:01:01 | 5.5% | 6.5% | 4% |
| 51:01:01 | 1.5% | 6.5% | 11% |
| 55:01:01 | 1.5% | 3% | 2% |
| 57:01:01 | - | 2% | 2% |
| 57:03:01 | 1.5% | - | - |
| 58:01:01 | 1.5% | 3.5% | 2% |
| 73:01:01 | - | 1% | - |
| ***HLA-C**** |  |  |  |
| 01:02:01 | - | 1% | 7.5% |
| 02:02:02 | - | 2% | 10% |
| 03:02:02 | 1.5% | - | 2% |
| 03:03:01 | 1.5% | 6.5% | 2% |
| 03:04:01 | 11.5% | 2% | 2% |
| 04:01:01 | 8% | 9% | 11% |
| 05:01:01 | 7% | 4.5% | 7.5% |
| 06:02:01 | 8% | 13% | 5.5% |
| 07:01:01 | 33% | 25.5% | 17.5% |
| 07:02:01 | 11.5% | 6.5% | 2% |
| 07:18:01 | 1.5% | 3% | - |
| 08:02:01 | 1.5% | 1% | 4% |
| 12:03:01 | 8% | 8.5% | 15.5% |
| 14:02:01 | 4% | 1% | **-** |
| 14:03:01 | - | 1% | 2% |
| 15:02:01 | - | 4.5% | 5.5% |
| 15:05:02 | - | 3% | 2% |
| 16:01:01 | 1.5% | 2% | 2% |
| 16:02:01 | 1.5% | 1% | - |
| 16:04:01 | - | 1% | - |
| 17:01:01 | - | 3% | 2% |
| 18:02:01 | - | 1% | - |
| ***HLA class II*** | | | |
| ***HLA-DRB1**** |  |  |  |
| 01:01:01 | - | 6.5% | 2% |
| 01:02:01 | 1.5% | - | - |
| 03:01:01 | 45.5% | 37% | 33% |
| 04:01:01 | 15% | 7% | 11% |
| 04:02:01 | 4% | 13% | 11% |
| 04:04:01 | 3% | 3% | - |
| 04:05:01 | 1.5% | 6.5% | 5.5% |
| 07:01:01 | 5.5% | 8.5% | 10% |
| 08:01:01 | 4% | 1% | 2% |
| 09:01:02 | - | - | 4% |
| 11:01:01 | 1.5% | 2% | 2% |
| 11:03:01 | - | 2% | 2% |
| 11:04:01 | 1.5% | 1% | - |
| 12:01:01 | - | 3% | 2% |
| 13:01:01 | 3% | 2% | - |
| 13:02:01 | 4% | 3% | 5.5% |
| 15:01:01 | 3% | - | - |
| 16:01:01 | 7% | 4.5% | 10% |
| ***HLA-DQA1**** |  |  |  |
| 01:01:01 | 1.5% | 6.5% | 2% |
| 01:02:02 | 13.5% | 7% | 15.5% |
| 01:03:01 | 3% | 2% | - |
| 01:63:01 | 1.5% | - | - |
| 02:01:01 | 5.5% | 8.5% | 10% |
| 03:01:01 | 25% | 27% | 20.5% |
| 03:02:01 | - | - | 4% |
| 03:03:01 | - | 3% | 7.5% |
| 04:01:01 | 3% | 1% | 2% |
| 05:01:01 | 44.5% | 39% | 33% |
| 05:05:01 | 1.5% | 6% | 5.5% |
| ***HLA-DQB1**** |  |  |  |
| 02:01:01 | 44.5% | 40% | 34.5% |
| 02:02:01 | 4% | 7% | 7.5% |
| 03:01:01 | 1.5% | 6% | 10% |
| 03:02:01 | 25% | 29% | 23% |
| 03:03:02 | 1.5% | 1% | 5.5% |
| 04:02:01 | 3% | 1% | 2% |
| 05:01:01 | 4% | 6.5% | 2% |
| 05:02:01 | 7% | 4.5% | 10% |
| 06:02:01 | 1.5% | - | - |
| 06:03:01 | 3% | 2% | - |
| 06:04:01 | 4% | 3% | 5.5% |
| ***HLA-DPA1**** |  |  |  |
| 01:03:01 | 75% | 83% | 81% |
| 01:04:01 | 1.5% | - | 2% |
| 02:01:02 | 22% | 15% | 17% |
| 02:02:02 | 1.5% | 2% | - |
| ***HLA-DPB1**** |  |  |  |
| 01:01:01 | 12.5% | 9% | 5.5% |
| 02:01:02 | 25% | 19% | 11% |
| 02:02:01 | 1.5% | 1% | - |
| 03:01:01 | 11.5% | 9% | 27% |
| 04:01:01 | 26% | 35% | 32.5% |
| 04:02:01 | 4% | 4.5% | 2% |
| 05:01:01 | 1.5% | 1% | 2% |
| 06:01:01 | - | 1% | - |
| 09:01:01 | 1.5% | 2% | 4% |
| 10:01:01 | 4% | - | - |
| 11:01:01 | 1.5% | 1% | - |
| 13:01:01 | - | 2% | 4% |
| 14:01:01 | 4% | - | 4% |
| 15:01:01 | 1.5% | - | 4% |
| 17:01:01 | - | 2% | - |
| 23:01:01 | - | 3% | 2% |
| 104:01:01 | 5.5% | 8.5% | 2% |
| 116:01 | - | 1% | - |
| 124:01:01 | - | 1% | - |
